# Limited Australian clinical guidelines for pain assessment in hospitalized older adults with cognitive impairment: implications for safe and quality care

**DOI:** 10.3389/fpain.2026.1895602

**Published:** 2026-07-29

**Authors:** Rosemary Saunders, Kaoru Nosaka, Seng Giap Marcus Ang, Natasya Raja Azlan, Aaron Alejandro, Rohini Sharma Bhardwaj, Olivia Gallagher, Irene Ngune, Amineh Rashidi, Yan Hui Celestine Wee, Nilufeur McKay

**Affiliations:** 1 Centre for Research in Aged Care, School of Nursing and Midwifery, Edith Cowan University, Joondalup, WA, Australia; 2 School of Health & Clinical Sciences, The University of Western Australia, Perth, WA, Australia; 3 School of Nursing, Faculty of Health Sciences, Curtin University, Perth, WA, Australia; 4 Alice Lee Centre for Nursing Studies, Yong Loo Lin School of Medicine, National University of Singapore, Singapore, Singapore

**Keywords:** clinical practice guidelines, cognitive impairment, dementia, hospital, nurses, pain assessment, safety and quality, scoping review

## Abstract

**Introduction:**

Pain assessment remains challenging in healthcare settings in patients who are unable to self-report their pain. More than 50% of individuals with dementia and other forms of cognitive impairment (CI) experience pain daily. However, pain is frequently overlooked and inadequately assessed by healthcare professionals due to their lack of knowledge and guidelines to inform pain assessment. Pain is also a leading cause of hospital presentation and inappropriate pain assessment can impact on pain management and compromise safe and quality care. Evidence-based guidelines that inform appropriate pain assessment and management are therefore essential. This scoping review aimed to explore pain assessment guidelines for patients with CI in hospital through an Australian focused search to inform pain assessment practice within our local context and subsequent research. The objectives were to identify accessible pain assessment guidelines for hospitalized older adults with CI and summarize and describe their characteristics.

**Methods:**

A scoping review was conducted, using the Joanna Briggs Institute methodology and reported in accordance with the PRISMA-ScR reporting framework. The search strategy was guided by the Population, Concept, and Context frameworks, focusing on older adults with CI, pain assessment guidelines, and hospital or acute care setting. The search was conducted in March 2026, using CINHAL, MEDLINE, Embase, Web of Science, Google, Yahoo Search, and Bing.

**Results:**

No Australian guidelines were located in the database search. Of 56 guidelines identified through internet searches, nine met the inclusion criteria and were included in data extraction. All nine included pain assessment indication; however, two provided no practical guidance, and the remaining seven offered only brief narrative descriptions of pain assessment steps. The most frequently recommended pain assessment tools in the guidelines for patients with CI were the Pain Assessment in Advanced Dementia and the Abbey Pain Scale. Discussion: By systematically mapping the extent, characteristics, and variability of guidelines for pain assessment of patients with CI guidelines, it is an important first step in driving innovation in pain assessment for older adults in Australia. It provides transferable insights that can inform the development and adaptation of international clinical guidelines. Scoping Review Registration (OSF):https://doi.org/10.17605/OSF.IO/HJSMZ

## 1 Introduction

The global population is aging at an unprecedented rate, with projections indicating that by 2030, one in six people worldwide will be aged 60 years or older, increasing the need for safe and quality care for older adults (1). Dementia presents a growing public health challenge; approximately 57 million people are living with dementia globally, nearly 10 million new cases diagnosed every year, and it is now a leading cause of death worldwide (2). Australia reflects these global aging trends. As of June 2020, 4.2 million Australians were aged 65 years and over, representing 16% of the total population which is projected to increase to between 21% and 23% of the total Australian population by 2066 (3). The prevalence of dementia in Australia is also increasing, with an estimated 425,000 Australians living with dementia in 2024, and projected to more than double to approximately 1.05 million by 2065. Dementia was the leading cause of death in Australia in 2023 (4).

Pain is recognized as a global health priority and for older people it is estimated to be double the prevalence of younger people less than 60 years (5). Over 50% of older adults with dementia and other causes of cognitive impairment (CI) experience pain daily and this impacts their activities of daily living and quality of life (6), however, it is often overlooked and not appropriately assessed by health professionals (7). Improving pain assessment and management is aligned with the World Health Organization (WHO) efforts to strengthen equitable access to quality care, contributing directly to the UN Sustainable Development Goal (SDG) 3 (8), which aims to promote well-being for all at all ages (9); as well as the WHO's Integrated care for older people approach (ICOPE) (10) and the United Nations Decade of Healthy Ageing (2021–2030) (11).

In Australia, pain management is a national health priority, reflected in the 2021 National Strategic Action Plan for Pain Management (NSAPPM) (12). Goal three of this plan emphasizes supporting health practitioners with evidence-based information and skills to deliver effective pain care (12). To achieve this, the National Strategy for Health Practitioner Pain Management Education was released in 2023, highlighting the need to improve pain assessment and management competencies for better understanding and treatment of pain (13).

Pain is also among the most common reasons for hospital presentation in Australia. A recent study identified a pain prevalence of 55.2% in adults presenting to urban Australian emergency departments (EDs) (14) and another study reported a 51% pain prevalence across 75 large public hospitals in a state of Australia (15). Despite its high prevalence, pain in older adults with CI is frequently poorly assessed and undertreated (16, 17), and longer times to analgesia in EDs have been reported in older adults than in younger age groups (14). One study on abdominal pain care in a United Kingdom ED also confirmed that older adults experienced prolonged delays in initiating pain management (18). Inadequate pain assessment undermines care quality, worsens clinical outcomes, and compromises patient dignity. It is associated with functional decline, increased risk of falls, sleep disturbance, behavioral symptoms, and psychological issues such as delirium and depression (19, 20).

Guidelines for standardizing and enhancing timely and appropriate assessment and management of pain are essential to healthcare providers' evidence-based quality care delivery (12). They are especially necessary when caring for patients with CI who are unable to communicate effectively and where pain may be under-recognized (21–23). Existing literature has reported that health professionals' adherence to guidelines remains inconsistent globally across various conditions and levels of care (24, 25). Herr et al. (26) provides Clinical Practice Recommendations in support of the American Society for Pain Management Nursing guidelines for pain assessment in the patient unable to self-report. Routine, repeated, and well-documented pain assessment is emphasized as an ethical imperative in healthcare, asserting that regardless of a patient's ability to self-report, pain assessment is essential to support multimodal treatment and effective communication among healthcare professionals (27). To address the challenges of assessing pain in populations unable to self-report, they advocate for comprehensive assessment strategies. Specifically, they recommend the Hierarchy of Pain Assessment as a clinical practice framework for all populations, including older adults with advanced dementia, and the use of behavioral assessment tools (27). The Assessment of Pain in Older People: UK National Guidelines (22) also recommend the use of appropriate pain assessment tools, evidence-based guidelines, and pain education for health professionals. Despite the importance of pain assessment guidelines in healthcare, it remains unclear whether existing Australian guidelines are evidence-based and address all aspects of pain assessments for adults, including guidelines for older adults unable to self-report their pain levels. The primary aim of this scoping review was to explore existing Australian pain assessment guidelines for hospitalized older adults with CI. Specific objectives to achieve this aim were to: (1) identify the existing and accessible pain assessment guidelines for hospitalized older adults with CI, and (2) summarize and describe characteristics of the existing guidelines for pain assessment in hospitalized older adults with CI.

## 2 Methods

### 2.1 Study design

Scoping reviews have been reported to provide a comprehensive overview of evidence, including the identification of existing guidelines (28). Our review methods followed the Joanna Briggs Institute (JBI) framework for scoping reviews (29, 30) and the Preferred Reporting Items for Systematic Reviews and Meta-analysis Extension for Scoping Reviews (PRISMA-ScR) (31).

In addition to a systematic database search of available evidence, this scoping review included a search of grey literature for guidelines published by Australian-based institutions and organizations. As guidelines are not always published in searchable or peer-reviewed journal articles, grey literature was considered a critical source. Our grey literature search was limited to Australia to provide a clear picture of guidelines available to inform pain assessment practice within our local context in subsequent national-specific research. We determined that a limited-location search would reduce algorithmic biases and improve the manageability and reliability of internet searches. A MEDLINE and Google search identified no systematic or scoping reviews on this topic.

### 2.2 Protocol

The review was registered with the Open Science Framework (OSF) on 26 February 2024 and updated March 2026 (32). An *a priori* review protocol was also registered with the OSF as an open material (https://doi.org/10.17605/OSF.IO/2DXM7).

### 2.3 Eligibility criteria

In this review, the population was older adults (aged 65 years or older) with CI, the concept refers to pain assessment guidelines, and the context was hospital or acute care setting. The search included primary studies and grey literature which report on pain assessment guidelines for older adults with CI in hospital or an acute care setting were included. In addition, the search was limited to studies published between 2021 and 2026, in English, but not restricted by country of origin. This date range was selected in line with the Australian National Health and Medical Research Council (33) guidance which recommends a maximum of five years for Clinical Practice Guideline (CPG) validity. We excluded pain assessment guidelines in nursing homes, community settings, long-term care, as well as publications and information relating to pain assessment tool validations, as they are outside the scope of this review.

Guidelines for the purpose of this review were defined as written clinical information informing pain assessment, either a CPG, protocol, policy, or tool(kit), and either stand-alone documents or embedded within a document. This is in alignment with the Australian Department of Health definition of “systematically developed statements to assist practitioners with decisions about appropriate health care for patients in specific circumstances” (34).

### 2.4 Information sources and search strategy

The search terms included *Pain assessment, Pain measurement, Guideline, Policies, Protocols, Tool, Cognitive impairment, Dementia,* and *Nonverbal*. The term “Tool” was intentionally incorporated to capture guidelines focused on the use of pain assessment tools for older adults with CI. The search was conducted between 4 and 18 March 2026 by KN and RS.

#### 2.4.1 Database search strategy

A systematic search of the literature was conducted across four electronic databases: MEDLINE EBSCOhost, CINAHL Ultimate EBSCOhost, Embase, and Web of Science. The search strings for each database are presented in Supplementary Material 1.

#### 2.4.2 Grey literature search strategy

The grey literature search strategy was designed to maximize retrieval of guidelines as standalone documents or embedded within broader clinical guidelines. It incorporated two search strategies: (1) search results generated when the search strategy was used (also known as organic search), using three major search engines: Google, Yahoo Search, and Bing, (2) a search targeting local organization websites, including state and federal health department websites, peak bodies and professional association websites/web sources related to pain, CI, and/or dementia in Australia. Peak bodies and professional associations that were included are Pain Australia, the Australian Pain Society, Chronic Pain Australia, the Australian Pain Management Association, Pain Nurses Australia, the Alzheimer's Association, Dementia Australia, and Dementia Training Australia.

To ensure the rigor of the grey literature search, the grey literature search steps proposed by Godin et al. (35) were followed, and a research librarian was consulted. Two search operators were used to specify guidelines and relevant information published by the Australian state/federal governments or Australian organizations: “site:gov.au” and “site:org.au”. The first 100 results (as recommended by the subject librarian) of each search hit were reviewed: the title and snippet were examined first, followed by the full content when the title and snippet were vague. Godin et al. (35), who state that the 100 hits encompass the most relevant hits and feasible numbers to screen. Furthermore, in this review, to avoid missing relevant results, it was also confirmed that in each search, the last 10 hits of the 100 found nothing relevant. The search strategy for the grey literature search is reported in Supplementary Material 2.

### 2.5 Selection of sources of evidence

The selection of sources of evidence was determined by whether they met the inclusion criteria for the protocol (36). Two hundred and five studies were located after duplicate removal through the database search and uploaded to Covidence (37). Following screening of abstract and title by RS and MA no studies met the criteria. The relevant grey literature search results were recorded in an Excel summary sheet, accompanied by screenshots of the web source containing guideline information. Along with the summary sheet, search dates, search strategies, search results, and notes were recorded in a separate search diary. The screenshots and the original contexts were initially blind screened by two reviewers (AA and RSB). When inclusion/exclusion conflicts between the initial reviewers were identified, they discussed the rationale behind their judgments with another author in this review group (RS) until all reviewers reached a complete consensus.

### 2.6 Data extraction

The JBI Manual for Evidence Synthesis (38) was used for data extraction of grey literature. Eighteen criteria were selected for data extraction informed by the AGREE II criteria (39), a standardized quality and reporting appraisal tool for clinical guidelines. The information relating to each criterion was extracted by two reviewers (NRA and AR) and recorded in an excel spreadsheet. These criteria included: (1) Title; (2) Author; (3) URL; (4) Target end user; (5) Target population; (6) Source of guideline; (7) Type of guideline; (8) Information for pain assessment for patients with CI; (9) Indication for pain assessment; 10) Pain assessment tools recommended for use in patients with CI; (11) Guideline for pain assessment practice; (12) Guideline for pain re-assessment practice; (13) Information about documentation requirements following assessment; (14) Supported by references; (15) Links to other resources; (16) Last updated; (17) Endorsement; and (18) Other information. Definitions were determined for each criterion to ensure standardization of the data extraction process (Supplementary Material 3). When data extracted by the two reviewers was not congruent, the reviewers revisited the records separately and confirmed or updated the extraction in discussion with another co-author (RS). This process continued until all reviewers agreed on the extracted data.

### 2.7 Synthesis of results

Results were synthesized using a descriptive approach with frequencies, based on the recommendations by Pollock et al. (30) and Tricco et al. (40). Supplementary Material 4 and Table 1 provide a summary table of the results.

**Table 1:** Pain assessment for older patients with CI.

Author/Year	Title	Information for pain assessment for patients with CI	Indication for pain assessment	Pain assessment tools recommended for use in patients with CI	Guideline for pain assessment practice	Guideline for pain re-assessment practice	Information about documentation requirements following assessment
Wong (2022) (41)	Pharmacological management of chronic non-cancer pain in frail older people	Embedded within document	Yes	Communicative patients: Brief pain inventory—short form, NRS	Not identified	Yes	No
				Non-communicative patients: Abbey Pain Scale, Electronic Pain Assessment Tool (ePAT), PAINAD, Doloplus-2 Scale			
Australian Commission on Safety and Quality in Health Care (2022) (42)	Opioid analgesic stewardship in acute pain clinical care standard—Acute care edition	Embedded within document	Yes	Faces Pain Scale, Abbey Pain Scale, PAINAD, PACSLAC, MOBID, Doloplus-2 Scale	Yes -Narrative procedural steps	Yes	Yes
NSW Ministry of Health (2022) (43)	Assessment and management of Behaviours and Psychological Symptoms associated with Dementia (BPSD): A handbook for NSW health clinicians providing services for people experiencing BPSD	Embedded within document	Yes	PAINAD, PACSLAC, Abbey pain scale	Yes-Narrative procedural steps	Not identified	No
NSW Agency for Clinical Innovation (2022) (44)	Fascia iliaca block guide: A method of preoperative pain management in older people with acute hip fractures (version 2)	Embedded within document	Yes	Abbey Pain Scale, PAINAD	Yes-Narrative procedural steps	Yes	Yes
Australian Commission on Safety and Quality in Health Care (2023) (45)	Hip fracture clinical care standard	Embedded within document	Yes	PAINAD, Abbey Pain Scale	Yes-Narrative procedural steps	Not identified	No
South Eastern Sydney Local Health District (2023) (46)	Acute pain management in the post anaesthetic care unit: Intravenous opioid pain protocol for adults fentanyl, hydromorphone (dilaudid), morphine and oxycodone	Embedded within document	Yes	PAINAD	Yes-Narrative procedural steps	Not identified	No
South Eastern Sydney Local Health District (2023) (47)	Wound—Managing Pain at Dressing Change	Embedded within document	Yes	PAINAD, Abbey Pain Scale	Yes-Narrative procedural steps	Not identified	Yes
Department of Health State Government of Victoria (2024) (48)	Pain in older people	Embedded within document	Yes	PACSLAC, PAINAD, Abbey Pain Scale	Yes-Assessment at rest and during activity	Yes	Yes
Canberra Health Services (2025) (49)	Canberra Health Services procedure acute pain services—Adult and paediatric	Embedded within document	Yes	PAINAD, Abbey Pain Scale	Not identified	Not identified	No

CI, cognitive impairment; NRS, the numeric rating scale; PAINAD, the pain assessment in advanced dementia; PACSLAC, the pain assessment checklist for seniors with limited ability to communicate; MOBID, mobilization-observation-behavior-intensity-dementia pain scale.

## 3 Results

**Figure 1:**
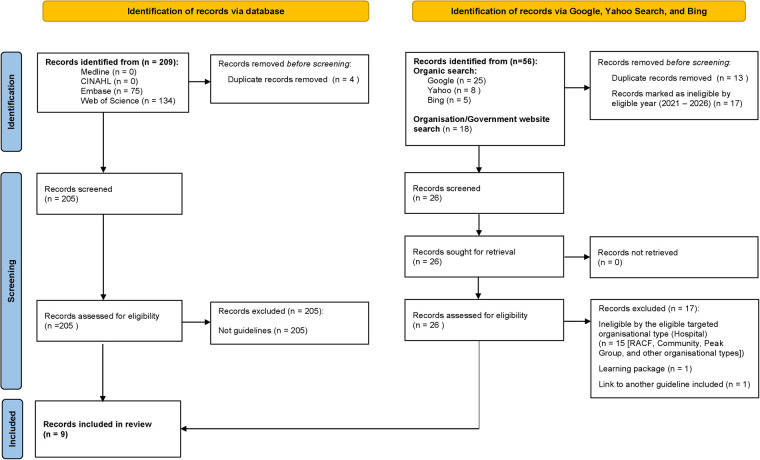
PRISMA-ScR flow diagram (50).

The database search yielded no results. The grey literature search located 56 records with information related to pain assessment in individuals with CI, but no stand-alone guideline. After removal of duplicates (*n* = 13) and records that were outside the criteria for the date range (*n* = 17), 26 records were screened for eligibility. Seventeen sources were subsequently excluded for the following reasons: ineligible organizational type (e.g., residential aged care facility, community, or peak bodies; *n* = 15), learning package (*n* = 1), and duplication through linkage to another included guideline (*n* = 1). A total of nine guidelines (41–49) were included, each addressing pain assessment of patients with CI in hospital (see Figure 1 for PRISMA flow diagram) (50).

### 3.1 Characteristics of included records (*n* = 9)

#### 3.1.1 Record characteristics

The included records were published or updated between 2022 and 2025. The majority of the records were produced by state governments or state health departments (*n* = 6) [New South Wales (4), Australian Capital Territory (1) & Victoria (1)] with two records from the Australian Commission on Safety and Quality in Health Care (ACSQHC) (Supplementary Material 4). One record (41) was a peer-reviewed journal article, identified via a search of peak body websites.

The primary target end users were stated in the resources as either healthcare professionals (HCPs) or clinicians (*n* = 6). Some records specifically described the targeted group such as nurses or midwives (e.g., post-anesthetic care unit registered nurses, registered nurses, nurse practitioners, enrolled nurses, registered midwives), as well as anesthetic medical officers, anesthetic consultants and registrars, and medical officers. The description of the target group varied across the records, with participants referred to as either older patients or older adults.

The records included guidelines (*n* = 2), clinical care standards (*n* = 2), standard operating procedures (*n* = 2), and a handbook, protocol, and toolkit (*n* = 1 each) (Supplementary Material 4). Most records (*n* = 6) were supported by references, including peer-reviewed literature and other clinical guidelines, and provided links to additional resources related to standard operating procedures, policies, procedures, guidelines, training and competencies (*n* = 7). More than half of the records (*n* = 5) were formally endorsed by the ACSQHC, state government committees [e.g., South East Sydney Local Health District (SESLHD) Drug and Therapeutics Committee] or peak professional organizations (e.g., Australian and New Zealand College of Anaesthetists & Faculty of Pain Medicine) (Supplementary Material 4).

### 3.2 Pain assessment for older patients with CI

The indications for undertaking a pain assessment and information were stated in all records (Table 1), but there were no “standalone” guidelines on pain assessment for patients with CI.

One record (41) had two sub-categories of cognitively impaired patients: communicative patients and non-communicative patients. For communicative patients, a brief pain inventory-short form and the Numeric Rating Scale (NRS) were recommended. The most recommended tool for patients with CI was the Pain Assessment in Advanced Dementia (PAINAD) scale (51) (*n* = 9) and the Abbey Pain Scale (52) (*n* = 8), followed by the Pain Assessment Checklist for Seniors with Limited Ability to Communicate (PACSLAC) (53) (*n* = 3), and Doloplus-2 (54) (*n* = 2). Other tools recommended were Electronic Pain Assessment Tool (ePAT) (55), Faces Pain Scale (56), and Mobilization-Observation-Behavior-Intensity-Dementia Pain Scale (MOBID) (57) (*n* = 1, each) (Table 1).

Direction or guidance on pain assessment practice was provided in seven records; however, none presented this information using flowcharts or other visual formats. Instead, six of these records (42–47) relied on brief narrative descriptions primarily only outlining procedural steps. The remaining record provided instructions for pain assessment conducted both at rest and during activity (48). Direction or guidance on repeat pain assessment following initial assessment was given in four of these seven records. Information about documentation requirements following assessment was provided in four records (42, 44, 47, 48) (Table 1).

One record (44) functioned as a guideline intended to be adapted to meet the needs of individual local hospitals and health districts, with the requirement that appropriate local policies and clinical governance frameworks should be established prior to implementation of a fascia iliaca block (FIB) as an analgesic option for patients with acute hip fracture. In contrast, the clinical care standard developed by the ACSQHC (42) and the online toolkit produced by the Victorian Department of Health (48) provided the most comprehensive and detailed guidance on pain assessment in people with CI. However, even these two resources did not provide clearly defined, step-by-step pain assessment processes that could be readily and consistently applied across all clinical settings or patient groups.

## 4 Discussion

The aim of this review was to explore existing Australian pain assessment guidelines for hospitalized older adults with CI. While pain assessment of cognitively impaired older adults is widely researched (55, 58–60), to our knowledge, this is the first scoping review to adopt a nationwide approach. The review contributes a structured summary of Australian guidelines that include information about pain assessment for patients with CI rather than evaluating individual assessment instruments. Thereby providing insights for international audiences seeking to strengthen pain assessment governance and implementation across health systems. The database search did not provide any Australian specific published guidelines. Nine resources were identified in the grey literature search that provide some tailored guidance (within guidelines that target specific conditions or care) for pain assessment of patients with CI. Therefore, the main finding of this scoping review is the absence of a standalone Australian clinical guideline for pain assessment in hospitalized older adults with CI. This indicates a significant gap with implications for safe, consistent, and quality care of older people. From an international perspective, the American Society for Pain Management Nursing (ASPMN) 2024 position statement and accompanying clinical practice recommendations provide guidance for pain assessment in patients unable to self-report (26), including neonates, infants, toddlers and young children; intellectually disabled (ID), critically ill/unconscious; traumatic brain injury or stroke; advanced dementia; and end of life. It also provides a useful algorithm to guide decision-making when assessing pain in those unable to self-report. However, there remains a notable absence of a stand-alone clinical guideline for pain assessment in hospitalized older adults with CI that considers assessment of all factors and especially atypical symptom presentation and behavioural indicators, baseline cognitive function, environmental factors (such as noise, lighting, and unfamiliar surroundings that may exacerbate confusion), and the contribution of family or caregivers in recognising and interpreting pain behaviours (61).

It is known that the implementation of clinical guidelines in busy healthcare settings is challenging (24). For guidelines to have clinical appropriateness and utility at the bedside, they need to include clear diagrammatic or algorithmic displays to ensure clinician uptake (62). In the resources located in this study, none used flow diagrams or other visual formats to guide pain assessment for older adults with CI in hospital, and few provided guidance on pain reassessment after initial assessment or treatment. Failing to assess pain can cause severe adverse outcomes and is recognized as a preventable patient safety adverse event (63). To mitigate this risk, guidelines need to be designed that are easy to incorporate into clinical information systems, and electronic medical records as part of real time clinical practice (62). Clinical decision support systems can also include information from guidelines to provide alerts to standardize pain assessments, trigger appropriate tool use, and alerts for reassessment (64). Future guideline development should also consider integration into electronic medical record systems and clinical decision-support tools to support standardized pain assessment, reassessment prompts, pain documentation, and timely analgesic management practices.

Nurses and other health professionals are responsible for effectively assessing and managing pain, and evidence-based clinical guidelines and resources are required to support clinicians' practice and education. The Australian National Strategy for Health Practitioner Pain Management Education Resource identifies the need for appropriate pain assessment of older Australians with CI (13). However, some information embedded within other guidelines was fragmented and may not adequately inform the delivery of safe and quality care for pain management. It is reported that an absence of clinical guidelines and inadequate health professionals' knowledge are barriers to appropriate pain assessment in patients with CI (65, 66). Also, longer times to analgesia have been reported in older adults in emergency departments than in younger age groups as an outcome of clinicians and system errors including under assessment of pain (14). Another key finding in this review is the overall insufficiency of evidence-based information and adherence to the AGREE II criteria we extracted (39) which further reinforces the need for the development of robust evidence-based pain management guidelines for older adults with CI. Pain assessment practices should be universal, and there is a need for clear guidelines globally and nationally to support pain assessment for older adults for better pain management. The results of the study will inform the development of guidelines in Australia, and these findings also have international relevance.

### 4.1 Implications

Given the increasing prevalence of CI and dementia in the aging Australian and global populations, it is crucial that guidelines are developed to support appropriate pain assessment practices to facilitate effective pain management. This is especially important because inaccurate assessment and the reluctance to provide analgesia to an older adult with CI, due to the concern of worsening delirium or sedation, leads to untreated pain (67). Mahama and colleagues (68) completed a scoping review on management approaches among patients with dementia in an acute hospital setting which identified several validated instruments to assess and manage pain in patients with dementia such as PAINAD. The review also found that validated tools alone were not adequate to change practice. This further highlights the critical need for not only further research on evidence-based clinical guidelines but also policy and education to inform implementation strategies that will reach the bedside for improved safety and quality care. Despite being situated within an Australian context, the findings of this review have clear relevance for an international audience and reinforce the importance of pain assessment for older adults and those with CI. The findings of this review may trigger clinicians from other countries to consider what guidelines are available and how they are a support or barrier to consistent pain assessment practices of older adults in hospitals and other settings, and how they translate to sustainable routine clinical practice.

### 4.2 Strengths and weaknesses

This scoping review has demonstrated that pain assessment guidelines for older adults with CI in hospitals are lacking in Australia, and their development is urgently required. Our findings can inform future development and improvement projects of pain assessment and management guidelines.

There are also some limitations to this review. Our grey literature search was limited to publicly accessible records thus records on private organizations' intranet systems may not have been captured limiting comprehensiveness of our scoping review. The search was also limited to guidelines for hospital practice, which aligned with our scope and aims; however, it excluded guidelines used in aged-care facilities, community, or other non-hospital organizations. We also acknowledge this review focuses on Australian specific literature which limits the analysis of international guidelines.

## 5 Conclusion

By highlighting the insufficient tailored clinical guidance for pain assessment of patients with CI in hospital in Australia, we hope this is a call to action for national guidelines to be developed; and encourages consideration of international guideline development. Future guideline development should incorporate behavioral pain assessment approaches, interdisciplinary collaboration and integration into clinical workflows and electronic medical record systems to improve care quality and patient outcomes. The development of evidence-based clinical guidelines for pain assessment of patients with CI can optimize clinical decision making for effective pain management and ultimately improve care for older patients who are unable to self-report their pain.
