# Supplementary material for: Limited Australian clinical guidelines for pain assessment in hospitalized older adults with cognitive impairment: implications for safe and quality care
Source: Front Pain Res (Lausanne). 2026 Jul 29;7:1895602. doi: 10.3389/fpain.2026.1895602 (PMC13461886; doi:10.3389/fpain.2026.1895602)
Supplement: Supplementary file 1 [file Table1.docx]

**Supplementary Material 1.** Search strategies for each database

| **Database** | **Search strategy** |
| --- | --- |
| Database: CINAHL Ultimate  Host: EBSCO  Proximity search | (pain assess* OR pain measur*) n4 (guideline* OR protocol*OR tool*OR polic*), (cognitive impairment*OR dementia OR nonverbal*) AND Australia* |
| Database: MEDLINE  Host: EBSCO  Proximity search | (pain assess* OR pain measur*) n4 (guideline* OR protocol*OR tool*OR polic*), (cognitive impairment*OR dementia OR nonverbal*) AND Australia* |
| Database: EMBASE  Host: EMBASE  Proximity search | ('pain assess*' OR ('pain measur*' NEAR/4 (guideline OR tool* OR polic*))) AND ('cognitive impairment'/exp OR 'cognitive impairment' OR 'dementia'/exp OR dementia OR 'nonverbal' OR (non AND verbal)) AND english:la AND australia* |
| Database: Web of Science  Host: Web of Science  Proximity search refined by Web of Science Categories | (TS=(pain assess* OR pain measur* Near/4 guideline* OR protocol* OR tool* OR polic*)) AND TS=(cognitive impairment OR dementia OR nonverb al or (Non AND verbal)) AND TS=(Australia*) |
