# Supplementary material for: Limited Australian clinical guidelines for pain assessment in hospitalized older adults with cognitive impairment: implications for safe and quality care
Source: Front Pain Res (Lausanne). 2026 Jul 29;7:1895602. doi: 10.3389/fpain.2026.1895602 (PMC13461886; doi:10.3389/fpain.2026.1895602)
Supplement: Supplementary file 2 [file Table2.docx]

**Supplementary Material 2.** Search strategies for each internet browser

| **Search engine** | **Search strategy** |
| --- | --- |
| Organic search via Google, Yahoo Search, and Bing | (pain assess* OR pain measur*), (guideline* OR protocol*OR tool*OR polic*), (cognitive impairment*OR dementia OR nonverbal*) site:gov.au  (pain assess* OR pain measur*), (guideline* OR protocol*OR tool*OR polic*), (cognitive impairment*OR dementia OR nonverbal*) site:org.au |
| Organisational document review via Google | Online organisational documents obtained through federal/state/regional health services websites |
| Organisational document review via Google | Online organisational documents obtained through Australian Peak bodies & Professional associations related to Pain |
| Organisational document review via Google | Peak bodies & Professional associations related to Cognitive Impairment & Dementia |
