# Supplementary material for: Limited Australian clinical guidelines for pain assessment in hospitalized older adults with cognitive impairment: implications for safe and quality care
Source: Front Pain Res (Lausanne). 2026 Jul 29;7:1895602. doi: 10.3389/fpain.2026.1895602 (PMC13461886; doi:10.3389/fpain.2026.1895602)
Supplement: Supplementary file 3 [file Table3.docx]

**Supplementary Material 3.** Characteristics of Records (N = 9)

| **Author/Year** | **Title** | **Source of guideline** | **Target end user** | **Target population** | **Type of guideline** | **Supported by references** | **Links to other resources** | **Endorsement** |
| --- | --- | --- | --- | --- | --- | --- | --- | --- |
| Wong (2022) (41) | Pharmacological management of chronic non-cancer pain in frail older people | Other - Journal Article | HCPs | Older patients | Guideline | Yes | Yes | No |
| Australian Commission on Safety and Quality in Health Care (2022) (42) | Opioid analgesic stewardship in acute pain clinical care standard – Acute care edition | National Authority / Government Agency  ACSQHC | Clinicians including Doctors, Dentists, Nurses, Midwives, Pharmacists, Nurse practitioners, Aboriginal and Torres Strait Islander health workers or practitioners, Podiatrists endorsed for scheduled medicines, Paramedics, Allied health practitioners | Patients of all ages with acute pain for whom opioid analgesics may be considered or prescribed | Clinical care standard | Yes | Yes | Yes  Australian and New Zealand College of Anaesthetists & Faculty of Pain Medicine, Australasian College for Emergency Medicine, ASCEPT, Australian College of Nurse Practitioners, Australian College of Nursing, Australian Orthopaedic Association, Children’s Healthcare Australasia, CENA, NPS MedicineWise, Pharmaceutical Society of Australia, RANZCOG, SHPA |
| NSW Ministry of Health (2022) (43) | Assessment and management of Behaviours and Psychological Symptoms associated with Dementia (BPSD): A handbook for NSW health clinicians providing services for people experiencing BPSD | Department of Health  NSW Ministry of Health | Clinicians | People living with dementia who exhibit BPSD | Handbook | Yes | Yes | No |
| NSW Agency for Clinical Innovation (2022) (44) | Fascia iliaca block guide: A method of preoperative pain management in older people with acute hip fractures (version 2) | Department of Health  NSW Agency for Clinical Innovation | HCPs | Older people with acute hip fractures | Guideline | No | Yes | Yes  NSW Agency for Clinical Innovation |
| Australian Commission on Safety and Quality in Health Care (2023) (45) | Hip fracture clinical care standard | National Authority / Government Agency   ACSQHC | HCPs | Patients aged 50 years and over presenting with a suspected or confirmed hip fracture, including those who sustain a hip fracture while in hospital | Clinical care standard | No | No | Yes  ACSQHC |
| South Eastern Sydney Local Health District (2023) (46) | Acute pain management in the post anaesthetic care unit: Intravenous opioid pain protocol for adults fentanyl, hydromorphone (dilaudid), morphine and oxycodone | Health Department  SESLHD | Anesthetic medical officers, Post anesthetic care unit-registered nurses | Post-surgery adult patients | Protocol | No | Yes | Yes  SESLHD Clinical and Quality Council |
| South Eastern Sydney Local Health District (2023) (47) | Wound – Managing Pain at Dressing Change | Health Department  SESLHD | RN, Medical officers, Nurse practitioners | Patients with wounds | Standard operating procedure | Yes | Yes | Yes  SESLHD Drug and Therapeutics Committee |
| Department of Health State Government of Victoria (2024) (48) | Pain in older people | Department of Health  Victorian Department of Health. | HCPs | Older people in hospital | Toolkit (website) | Yes | No | No |
| Canberra Health Services (2025) (49) | Canberra Health Services procedure acute pain services - Adult and paediatric | Hospital  Canberra Health Services | Anesthetic consultants, Anesthetic registrars, Medical officers, Nurses RNs, ENs and RMs who are competent in the procedure, Students under direct supervision | Adults and children with acute pain | Standard operating procedure | Yes | Yes | No |
| **Abbreviations:**  EN - Enrolled nurse, HCP - Healthcare professional, RM – Registered midwife, RN - Registered nurse  ACSQHC - Australian Commission on Safety and Quality in Health Care  ASCEPT - Australasian Society of Clinical and Experimental Pharmacologists and Toxicologists,  CENA - College of Emergency Nursing Australasia Ltd,  NSW – New South Wales  RANZCOG - The Royal Australian and New Zealand College of Obstetricians and Gynaecologists,  SESLHD - South Eastern Sydney Local Health District,  SHPA - The Society of Hospital Pharmacists of Australia | | | | | | | | |
