# Supplementary material for: Limited Australian clinical guidelines for pain assessment in hospitalized older adults with cognitive impairment: implications for safe and quality care
Source: Front Pain Res (Lausanne). 2026 Jul 29;7:1895602. doi: 10.3389/fpain.2026.1895602 (PMC13461886; doi:10.3389/fpain.2026.1895602)
Supplement: Supplementary file 4 [file Table4.docx]

**Supplementary Material 3.** Definition of 18 Criteria

| **Criterion** | **Definition** |
| --- | --- |
| 1. Title | Record’s title |
| 2. Author | Author(s) who prepared the record |
| 3. URL | URL locating a record |
| 4. Target end user | The specific person or group intended to use a guideline. |
| 5. Target population | Target patient population of the guideline (e.g. palliative care, ED patients; patients who are unable to self-report the presence of pain; examples include patients with dementia, delirium, cognitive impairment or communication issue) |
| 6. Source of guideline | Guideline Source (hospital; Health Department, peak body, other-describe) |
| 7. Type of guideline | Guideline Type (e.g., clinical practice guideline, policy, position statement, protocol, standard operating procedure, other-describe) |
| 8. Information for pain assessment for patients with CI | Is the guideline specific to pain assessment for patients with CI? Yes (i.e. stand-alone document) or No (i.e. information embedded in other policies - state policy name) |
| 9. Indication for pain assessment | Indications for pain assessment stated |
| 10. Pain assessment tools recommended | Pain assessment tool(s) recommended for use in patients with CI (list tool name or 'Not identified') |
| 11. Guideline for pain assessment practice | Direction or guidance on pain assessment practice. e.g. Flowchart or procedural steps; assessment at rest and movement (note guidance or 'Not identified') |
| 12. Guideline for pain re-assessment practice | Direction or guidance on pain reassessment practice following intervention (note guidance or 'Not identified') |
| 13. information about documentation requirements following assessment | Information about documentation requirements following assessment. If Yes (state) or No |
| 14. Supported by references | Guideline is evidence based supported by references (Yes/No) |
| 15. Links to other resources | Links to other resources, e.g., related SOP, policies, procedures, guidelines, training & competencies |
| 16. Last updated | Date of last review or record as n/a if no date |
| 17. Endorsement | Endorsement. If Yes (state by who) or No |
| 18. Other information | Other Information |
